# Chromosome-level genome assembly of *Cnidium monnieri*, a highly demanded traditional Chinese medicine

**DOI:** 10.1038/s41597-024-03523-6

**Published:** 2024-06-22

**Authors:** Zixuan Wang, Jiaxin He, Qi Qi, Kaixuan Wang, Huanying Tang, Yimeng Feng, Xinyue Zhao, Shanyong Yi, Yucheng Zhao, Dingqiao Xu

**Affiliations:** 1 Department of Resources Science of Traditional Chinese Medicines, School of Traditional Chinese Pharmacy, China Pharmaceutical University, Nanjing, 210009 China; 2 Department of Biological and Pharmaceutical Engineering, West Anhui University, Lu’an, 237012 China; 3 Medical Botanical Garden, China Pharmaceutical University, Nanjing, 210009 China; 4 School of Pharmacy, Shaanxi University of Chinese Medicine, Xi’an, 712046 China

**Keywords:** Genome assembly algorithms, Bioinformatics

## Abstract

*Cnidium monnieri*, a medicinal herb of the *Cnidium* genus and the Apiaceae family, is among the most important traditional Chinese medicines and is widely distributed in China. However, to date, no *C. monnieri*-related genomic information has been described. In this study, we assembled the *C. monnieri* genome of approximately 1210.23 Mb with a contig N50 of 83.14 Mb. Using PacBio HiFi and Hi-C sequencing data, we successfully anchored 93.86% of the assembled sequences to 10 pseudochromosomes (2n = 20). We predicted a total of 37,460 protein-coding genes, with 97.02% of them being functionally annotated in Non-Redundant, Gene Ontology, Kyoto Encyclopedia of Genes and Genomes, and other databases. In addition, we identified 2,778 tRNAs, 4,180 rRNAs, 258 miRNAs, and 1,700 snRNAs in the genome. This is the first reported *C. monnieri* genome. Hopefully, the availability of this chromosome-level reference genome provides a significant basis for upcoming natural product-related biosynthetic pathway assessment in *C. monnieri*.

## Background & Summary

*Cnidium monnieri*, of the Apiaceae family, is among the most important traditional Chinese medicines. Commonly referred to as “She Chuang Zi”, it has been traditionally used for long in China, Korea, Vietnam, and Japan against various diseases. The first record of *C. monnieri* could be found in Shennong’s Classic of Materia Medica. The fruit of *C. monnieri* contains various active ingredients, including volatile oils, coumarins, chromones, glycosides, or terpenoids^1^, and it retains diverse (e.g., anti-osteoporotic, anti-adipogenic, and anti-fungal) properties^2–5^. However, inconsistent *C. monnieri* quality represents a persistent problem, limiting its widespread application and raising medication safety concerns. Phytochemical analysis revealed significant variations across different geographical regions in the type and content of coumarins^6,7^, the primary chemical constituents of *C. monnieri*. These variations could potentially result from a combination of external environmental factors and internal gene regulation. Due to genomic information scarcity, our understanding of the coumarin synthesis mechanism in *C. monnieri* remains incomplete, hindering our ability to effectively address the underlying causes contributing to significant variations across different geographical regions in the type and content of coumarins.

**Fig. 1:**
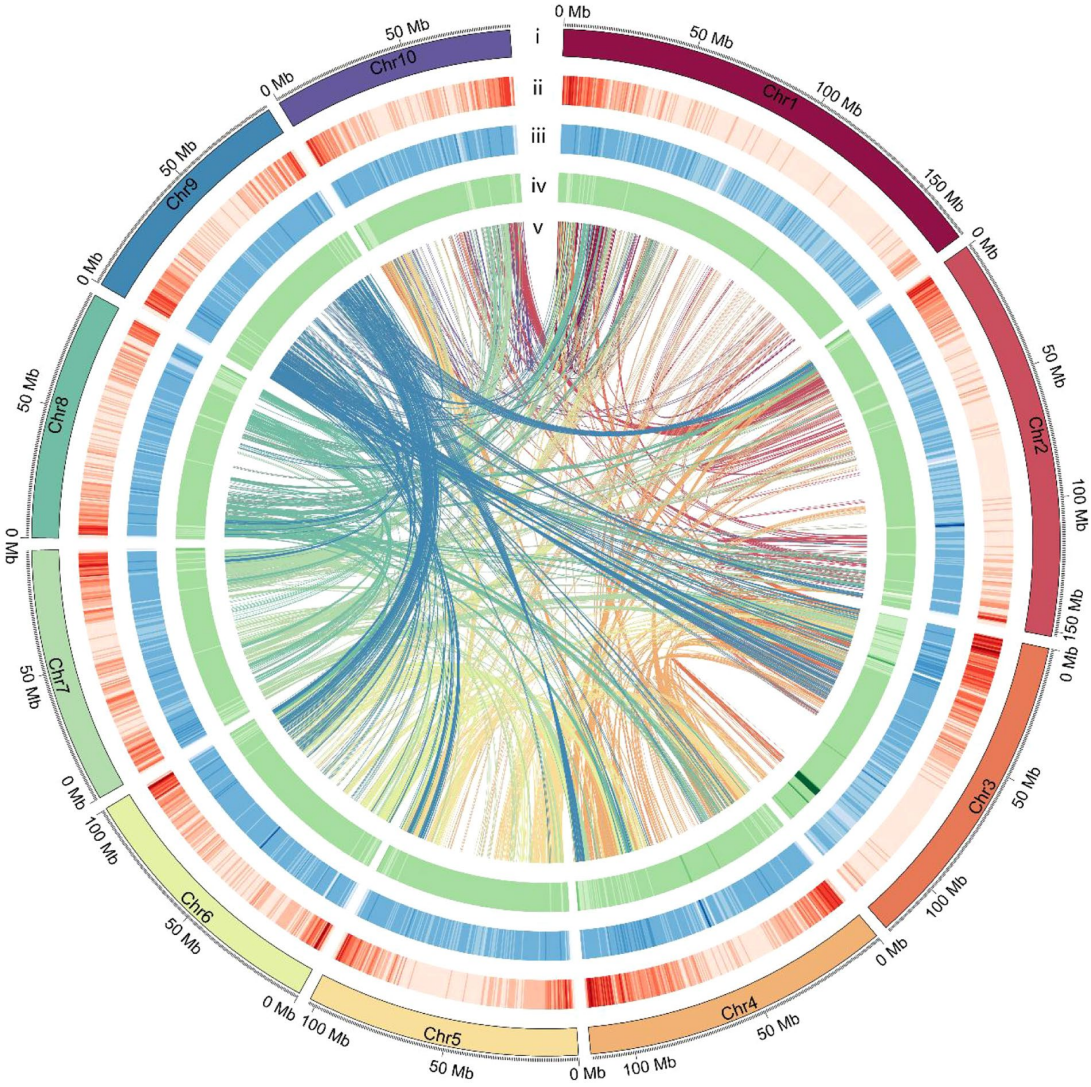
Genome characteristics of *C. monnieri*. Circos plot from the outer to the inner layers represents the following: (i) Pseudo-chromosomes (Chr1-Chr10); (ii) Gene density; (iii) The density of repeat sequences; (iv) GC content; (v) Each linking line in the center of the circus plot indicates a pair of homologous genes.

In this study, we used high-fidelity (HiFi) reads and high-throughput chromosome conformation capture (Hi-C) sequencing technologies to assemble the *C. monnieri* chromosome-level genome. We revealed a final genome size of 1,210.23 Mb with a scaffold N50 length of 83.14 Mb and successfully anchored 93.86% of the assembled genome sequences to 10 chromosomes (Fig. 1). We identified a total of 36,344 protein-coding genes, all of which were functionally annotated. Technological advancements contributed to the successful completion of several genome sequencing projects, such as those targeting *Agastache rugosa*^8^, *Hibiscus syriacus*^9^, and *Rhododendron vialii*^10^. However, to date, no *C. monnieri*-related genome information has been made available. To the best of our knowledge, this study describes first the *C. monnieri* genome. We are convinced that this study will provide significant resources for investigating the biosynthetic mechanisms of this species.

## Methods

### Sample collection, library construction, and sequencing

We extracted high-quality genomic DNA from the aerial parts of *C. monnieri* using a modified cetyltrimethylammonium bromide (CTAB) method^11^ and samples collected from Fengtai County (32°33′N, 116°21′E), Anhui Province, China. We used a NanoDrop 2000 spectrophotometer (NanoDrop Technologies, Wilmington, DE, USA), a Qubit 3.0 Fluorometer (Life Technologies, Carlsbad, CA, USA), and 0.8% agarose gel electrophoresis to determine the concentration and quality of the extracted DNA samples.

We used the aerial parts of *C. monnieri* to construct a library for Hi-C analyses as described previously^12^. Briefly, we cross-linked the fresh tissue samples using 3% formaldehyde under vacuum infiltration at 4 °C for 30 min and quenched the cross-linking reaction with a final concentration of 0.375 Mb glycine for 5 min. Next, we lysed the cross-linked samples. We inactivated the endogenous nucleases with 0.3% SDS, then digested the chromatin DNA with 100 U of MboI (NEB), marked it with biotin-14-dCTP (Invitrogen), and ligated it using 50 U of T4 DNA ligase (NEB). After reversing the cross-links, we extracted the ligated DNA using the QIAamp DNA Mini Kit (Qiagen) according to the manufacturer’s instructions. We sheared the purified DNA into 300–500-bp fragments and further blunt-end repaired and A-tailed them, followed by adaptor supplementation, purification through biotin-streptavidin-mediated pull-down, and PCR amplification. Finally, we quantified and sequenced the Hi-C libraries using the MGI-seq platform (BGI, China), generating 850,286,793 raw reads and 255.09 Gb of raw bases. We trimmed the raw data using Trimmomatic with default parameters to truncate sequencing junctions and low-quality fragments. Basic statistics on data quality after trimming using FastQC with default parameters show that 829,780,252 clean reads are generated, along with 247.76 Gb of clean bases, and a clean rate of 97.59%.

### RNA sequencing and analysis

We collected the stem, leaf, flower, and seed tissues of *C. monnieri* plants for RNA extraction. We extracted total RNA from the samples using TRIzol Reagent (Invitrogen, CA, USA) and verified RNA purity and integrity using a NanoDrop 2000 spectrophotometer (NanoDrop Technologies, Wilmington, DE, USA) and the Bioanalyzer 2100 system (Agilent Technologies, CA, USA). We assessed RNA contamination using 1.5% agarose gel electrophoresis. We used the BGISEQ sequencing platform to obtain RNA sequencing information for *C. monnieri*. We trimmed raw reads using SOAPnuke (v2.1.0)^13^, aligned clean reads to the reference genome using HISAT2 (v2.2.1)^14^ with default parameters and retained only uniquely mapped reads. We estimated the expression values using RSEM (v1.3.3)^15^ as fragments per kilobase of the exon model per million reads mapped (FPKM). We considered genes with FPKM > 0 expressed and used them for further analysis. We used DESeq2 (v1.22.2)^16^ to identify differentially expressed genes (DEGs) using FDR < 0.05 and (log2FC > 1 || log2FC < −1).

### Genome assembly

We obtained raw reads using DNBSEQ. We subjected the T7 platform to quality control using FastQC and Trimmomatic to filter out adapter sequences and low-quality reads. The initial assembly of the contig genome using Hifiasm (v0.19.5)^17,18^ resulted in a total length of 1658.64 Mb with a Contig N50 of 78.45 Mb. After using Hi-C scaffolding, the final genome assembly yielded a total length of 1210.23 Mb with a Contig N50 of 83.14 Mb (Table 1).

**Table 1:** Assembly statistics of *C. monnieri*.

Assembly	test
anchor ratio (%)	93.8594
Assembly length (bp)	1,210,229,788
BUSCO (%)	98.6
Contig N50 (bp)	83,135,773
HiFi reads coverage (%)	100.00
HiFi reads mapping rate (%)	99.73
Number of contigs	1,797
QV	57.1731

**Fig. 2:**
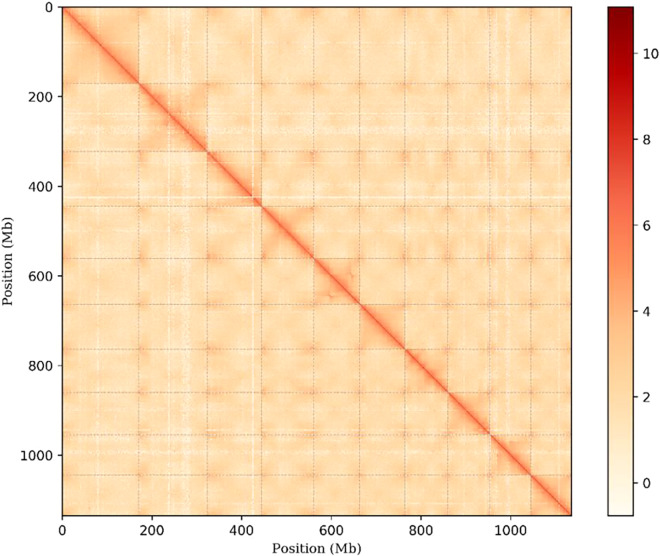
Whole genome Hi-C interaction heatmap (with a resolution of 250 kb) of *C. monnieri*. The strength of the interaction was represented by the color from yellow (low) to red (high).

We obtained the draft genome by assembling HiFi reads. We used Juicer (v1.6) to align the Hi-C reads to the draft assembly, subjected to quality control. We used 3D-DNA (v180922) to anchor primary contigs to the chromosomes. The final *C. monnieri* genome assembly was 1210.23 Mb with a scaffold N50 of 102.78 Mb. The Hi-C analyses scaffolded 10 pseudomolecules (Fig. 2), anchoring 93.86% of the *C. monnieri* genome assembly. The average *C. monnieri* genome assembly GC content was 34.93% (Fig. 1).

We evaluated the genome completeness using BUSCO (v5.2.2)^19^. Our analysis identified 98.6% (single-copy and duplicated genes: 95.1% and 3.5%, respectively), 0.2%, and 1.2% of the 1,367 predicted genes in this genome as complete, fragmented, and missing sequences, respectively (Table 2). These results suggested a highly complete assembled genome.

**Table 2:** BUSCO analysis of Species *C. monnieri* completeness.

Term	Genes	Percentage (%)
Total BUSCO groups searched	425	100
Complete BUSCOs	419	98.6
Complete and single-copy BUSCOs	404	95.1
Complete and duplicated BUSCOs	15	3.5
Missing BUSCOs	5	1.2
Fragmented BUSCOs	1	0.2

### Functional annotation of protein-coding genes

We inferred gene functions based on the best match of the alignments to the National Center for Biotechnology Information (NCBI) Non-Redundant, TrEMBL^20^, InterPro^21^, and Swiss-Prot^20^ protein databases using BLASTP (NCBI BLAST v2.11.0+)^22,23^ and the Kyoto Encyclopedia of Genes and Genomes database^24^ with an E-value threshold of 1E-5. We annotated the protein domains using PfamScan (pfamscan_version)^25^ and InterProScan (v5.50–84.0)^26^ based on the InterPro protein databases. We identified the motifs and domains within the gene models using PFAM databases^27^. We obtained Gene Ontology^28^ IDs for each gene using Blast2GO^29^. We functionally annotated approximately 97.02% of the predicted *C. monnieri* protein-coding genes with known genes, conserved domains, and Gene Ontology terms (Table 3).

**Table 3:** Functional annotated statistical results of *C. monnieri*.

	Number	Percent (%)
Total	37,460	
InterPro	26,452	70.61
GO	27,787	74.18
KEGG_ALL	35,502	94.77
KEGG_KO	12,383	33.06
Swissprot	25,357	67.69
TrEMBL	35,960	96.00
NR	36,259	96.79
Annotated	36,344	97.02
Unannotated	1,116	2.98

### Repetitive sequence annotation

To identify the repeated contents in the genome, we used two methods: homology-based and de novo prediction. In the homology-based analysis, we identified known TEs within the *C. monnieri* genome using RepeatMasker (v4.1.2)^30^ and the RepBase TE library^31,32^. We conducted RepeatProteinMask searches using the TE protein database as a query library. For de novo prediction, we constructed a de novo repeat library of the *C. monnieri* genome using RepeatModeler^33^ (http://www.repeatmasker.org/RepeatModeler/) and LTR-FINDER^33^, which could automatically execute two core de novo repeat-finding programs, RECON (v1.08)^34^ and RepeatScout (v1.0.5)^35^, to comprehensively conduct, refine, and classify the consensus models of putative interspersed repeats for the *C. monnieri* genome. Furthermore, we performed a de novo search for long terminal repeat (LTR) retrotransposons in the *C. monnieri* genome sequences using LTR_FINDER (v1.0.7)^33^. Moreover, we also identified tandem repeats using the Tandem Repeat Finder (TRF) package^36^ and non-interspersed repeat sequences, including low-complexity repeats, satellites, and simple repeats, using RepeatMasker. Finally, we merged the library files of the two methods, used a repeatmaker to identify the repeat content, and statistically analyzed the repeated sequence content predicted by the different software methods (Table 4).

**Table 4:** Repeat sequence statistics of *C. monnieri*.

Type	Repeat Size	% of genome
De novo	915,048,979	75.61
Proteinmask	192,958,977	15.94
Repeatmasker	212,676,540	17.57
Total	940,840,139	77.74
Trf	88,662,063	7.33

## Data Records

We deposited the hereby-described relevant data in the National Genomics Data Center (NGDC)^37,38^, Beijing Institute of Genomics, Chinese Academy of Sciences/China National Center for Bioinformation, under the BioProject accession number PRJCA022794, publicly accessible at https://ngdc.cncb.ac.cn/gwh. The BioSample accession ID is SAMC3313212. We deposited the genomic raw data in the Genome Sequence Archive (GSA) in NGDC under the accession number CRA014484, the *C. monnieri* genome project in the NCBI database under the BioProject accession ID of PRJNA1065623, the genome assembly at GenBank under the WGS accession ID of JBDIYB000000000^39^, the genomic raw sequencing data in the SRA at NCBI SRR28903605-SRR28903606^40,41^, and the transcriptomic raw sequencing data in the SRA at NCBI SRR27600471-SRR27600482^42–53^.

## Technical Validation

### Genome assembly quality evaluation

**Fig. 3:**
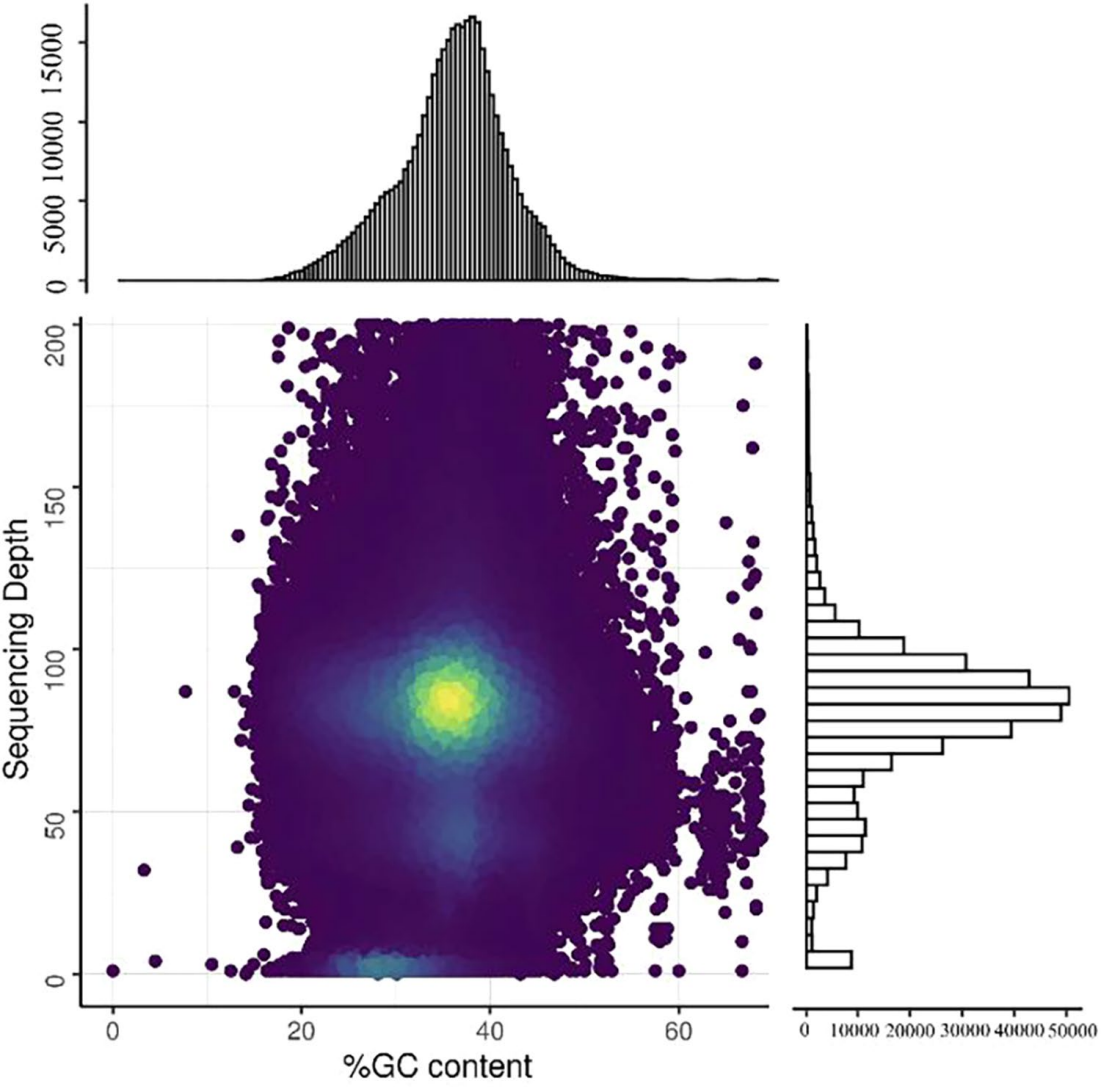
GC content and sequencing depth distribution density plot. The horizontal axis of the figure represents GC content, and the vertical axis represents sequencing depth. The contig coverage depth distribution is displayed on the right, and the GC content distribution is shown above. The large central plot is a scatter plot based on the GC content and coverage depth information of the contigs, where the color scale reflects the density of the plotted points.

To examine assembly integrity and sequencing uniformity, we aligned the HiFi reads in a final assembly using minimap2 (v2.21, parameters: HiFi: -ax map-hifi; ONT: -ax map-ont)^54^ and BWA^55^. We mapped a total of 99.73% of the raw reads. The average mapping and average sequencing depth was 79.5 (Table 5). Sequencing data could also be analyzed for GC bias and sample contamination using BWA and minimap2 software (Fig. 3). Moreover, we subjected the assembled genome to BUSCO^18^ using OrthoDB to evaluate genome completeness. Taken together, our BUSCO analysis revealed that 98.6%, 0.2%, and 1.2% of the 425 single-copy orthologs (in the Viridiplantae_odb10 database) were complete (single-copy and duplicated genes: 95.1% and 3.5%, respectively), fragmented, and missing, respectively (Table 2).

**Table 5:** Alignment statistics of *C. monnieri*.

data_type	Mapping rate (%)	Average sequencing depth	Coverage (%)	Coverage (> = 5X, %)	Coverage (> = 10X, %)	Coverage (> = 20X, %)
HiFi	99.73	79.05	100	95.58	94.62	94.16

To further evaluate the quality of the genome assembly, we used HiFi reads to assess the read-remapping ratio and coverage. We aligned the HiFi reads to the genome using the Juicer (v1.6) software with default parameters and the 3D-DNA (V180922) software for scaffolding. Our analysis revealed that the assembly displayed a high mapping rate and complete genome coverage of 99.73% and 100%, respectively, underscoring the high quality of the assembled genome (Table 5).

**Fig. 4:**
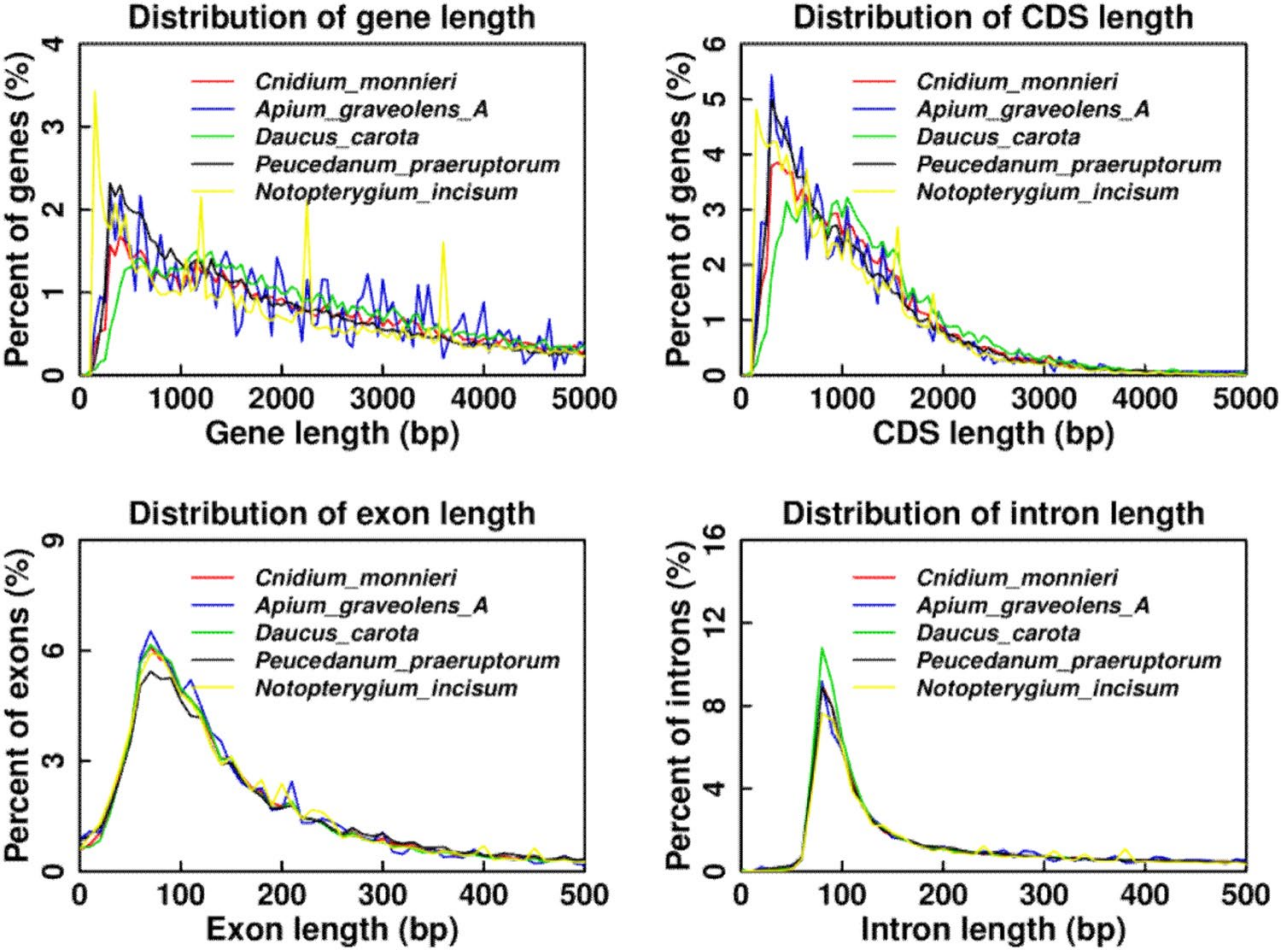
Annotated genes comparison of the distribution of gene length, CDS length, exon length, and intron length in *C. monnieri* with other closely related species. The x-axis represents the length and the y-axis represents the density of genes.

To determine prediction accuracy and reliability, we determined the distribution of gene length, CDS length, exon length, and intron length in *C. monnieri* and other closely related species (*Apium graveolens*^56^, *Daucus carota*^57^, *Peucedanum praeruptorum*^58^, and *Notopterygium incisum*^59^). The consistent distribution tendency among all species further supported the ideal annotated gene dataset for *C. monnieri* (Fig. 4).

## Data Availability

We followed the developers’ instructions for the bioinformatics tools used in this study. The software and code used are publicly accessible, with the version and parameters used specified in the Methods section. No custom code was used during the compilation of the dataset.
